# Selection of Stable Reference Genes for Quantitative RT-PCR Comparisons of Mouse Embryonic and Extra-Embryonic Stem Cells

**DOI:** 10.1371/journal.pone.0027592

**Published:** 2011-11-10

**Authors:** Kylee J. Veazey, Michael C. Golding

**Affiliations:** College of Veterinary Medicine and Biomedical Sciences, Texas A&M University, College Station, Texas, United States of America; Centro Cardiologico Monzino, Italy

## Abstract

Isolation and culture of both embryonic and tissue specific stem cells provide an enormous opportunity to study the molecular processes driving development. To gain insight into the initial events underpinning mammalian embryogenesis, pluripotent stem cells from each of the three distinct lineages present within the preimplantation blastocyst have been derived. Embryonic (ES), trophectoderm (TS) and extraembryonic endoderm (XEN) stem cells possess the developmental potential of their founding lineages and seemingly utilize distinct epigenetic modalities to program gene expression. However, the basis for these differing cellular identities and epigenetic properties remain poorly defined.

Quantitative reverse transcription-polymerase chain reaction (qPCR) is a powerful and efficient means of rapidly comparing patterns of gene expression between different developmental stages and experimental conditions. However, careful, empirical selection of appropriate reference genes is essential to accurately measuring transcriptional differences. Here we report the quantitation and evaluation of fourteen commonly used references genes between ES, TS and XEN stem cells. These included: *Actb*, *B2m*, *Hsp70*, *Gapdh*, *Gusb*, *H2afz*, *Hk2*, *Hprt*, *Pgk1*, *Ppia*, Rn*7sk, Sdha*, *Tbp* and *Ywhaz*. Utilizing three independent statistical analysis, we identify *Pgk1*, *Sdha* and *Tbp* as the most stable reference genes between each of these stem cell types. Furthermore, we identify *Sdha*, *Tbp* and *Ywhaz* as well as *Ywhaz*, *Pgk1* and *Hk2* as the three most stable reference genes through the *in vitro* differentiation of embryonic and trophectoderm stem cells respectively.

Understanding the transcriptional and epigenetic regulatory mechanisms controlling cellular identity within these distinct stem cell types provides essential insight into cellular processes controlling both embryogenesis and stem cell biology. Normalizing quantitative RT-PCR measurements using the geometric mean CT values obtained for the identified mRNAs, offers a reliable method to assess differing patterns of gene expression between the three founding stem cell lineages present within the mammalian preimplantation embryo.

## Introduction

During mammalian pre-implantation development a series of asynchronous divisions result in the formation of the blastocyst. At this stage of development three distinct cell types have emerged: the epiblast, trophectoderm and primitive endoderm, which give rise to the fetus, placenta and extraembryonic endoderm respectively [1]–[3]. To better define the developmental and transcriptional processes unique to each of these distinct lineages, *in vitro* cultured progenitor stem cells have been derived [4]–[7]. Analysis of ES, TS and XEN stem cell lines have revealed much about the cellular processes controlling mammalian development and demonstrated surprising differences in the epigenetic regulation of gene expression between these three lineages [7]–[12]. Identifying the biochemical factors underlying these differences remains an essential step to understanding the molecular processes driving development and better defining crucial aspects of mammalian stem cell biology.

Quantitative reverse transcription-polymerase chain reaction (qPCR) has emerged as a powerful technique to rapidly assess transcriptional differences between cell types and differing experimental conditions. However, accurate quantitative analysis is dependent upon proper, empirical selection of a suitable reference. Using published microarray data, and a novel statistical algorithm, (geNORM) Vandesompele and colleagues demonstrated that the geometric mean of three reference genes provided the most accurate and reliable means of normalizing qPCR expression data [13]. Subsequently, this experimental strategy has been validated and additional algorithms written and utilized to identify the most suitable reference genes for a variety of experimental conditions [14]–[25].

In this study we sought to identify a list of genes most suitable for use as normalization controls in qPCR-based comparisons between ES, TS and XEN stem cells or their *in vitro* differentiated progeny. In order to help identify candidate genes we set two main criteria that the mRNAs would have to fulfill: 1) the transcripts needed to be expressed above background and easily detectable, and 2) candidate mRNAs needed to be stably expressed between each of the three stem cell lineages under investigation. To this end we surveyed the recent literature and compiled a short list of fourteen candidate genes, including *Actb*, *B2m*, *Hsp70*, *Gapdh*, *Gusb*, *H2afz*, *Hk2*, *Hprt*, *Pgk1*, *Ppia*, Rn*7sK*, *Sdha*, *Tbp* and *Ywhaz* [14]–[28]. These genes belong to diverse functional classes and should not be co-regulated, thus providing a non-biased method of normalizing qPCR expression data.

To evaluate the stability of our candidate genes we isolated RNA from three independent lines of varying genotypes for each of the three stem cell types. This RNA was quantified and seeded into five independent qPCR reactions measuring each of the candidate genes. Using the geNORM, NormFinder and BestKeeper algorithms, we identify the *Pgk1*, *Sdha* and *Tbp* transcripts as the most stably expressed reference genes between each of these stem cell types. To determine which of these candidates was most suitable for use during *in vitro* differentiation studies, we cultured ES and TS cells in the absence of crucial growth factors LIF and FGF4 respectively. Using three independent RNA samples isolated on Day 0 and Day 8, we identify *Sdha*, *Tbp* and *Ywhaz* as well as *Ywhaz*, *Pgk1* and *Hk2* as the three most stable reference genes through the *in vitro* differentiation of ES and TS cells. Our results suggest that normalization of qPCR data using the geometric means of the transcripts listed above will yield the most accurate quantification of gene expression between these three unique stem cell types.

## Results

After a survey of the recent literature we curated a short list of fourteen commonly used reference genes and either designed new primers or pulled existing ones from references cited in the materials and methods. These genes are listed in Table 1 and represent several distinct functional classes so as to minimize the possibility of co-regulation. For each gene, a minimum of two independent primer sets were tested and of these, the primer set exhibiting the greatest efficiency was selected. To conduct an accurate survey of candidate gene expression levels between ES, TS and XEN stem cells we isolated RNA from at least three independent stem cell lines, representing at least two different genotypes. We postulate that utilizing lines derived from diverse genotypes will more accurately identify stable reference genes to be used in future studies contrasting patterns of gene expression.

**Table 1:** Descriptions of the fourteen candidate reference genes studied.

Symbol	Name	Accession	Brief Description
Rn7sk	7SK, small nuclear RNA	NR_030687	Small nuclear RNA that binds elongation factor P-TEFb and negatively regulates transcription.
Actb	Beta-Actin	NM_007393	A highly conserved protein found in all eukaryotic cells involved in various cellular processes such as cell motility and cytokinesis.
B2m	beta-2 microglobulin	NM_009735	Gene that codes for Beta-2-microglobulin, a component of the MHC
Gapdh	glyceraldehyde-3-phosphate dehydrogenase	NM_008084	Enzyme involved in metabolic and non-metabolic processes such as glycolysis, transcription activation, and apoptosis.
Gusb	glucuronidase, beta	NM_010368	Gene that codes for Beta-glucuronidase, which catalyzes the hydrolysis of B-D-glucuronic acid.
H2afz	H2A histone family, member Z	NM_016750	Member of the H2A histone family that is required for embryonic development.
Hk2	hexokinase 2	NM_013820	Enzyme that phosphorylates hexoses, including glucose to produce glucose-6-phosphate.
Hprt	hypoxanthine-phosphoribosyl transferase	NM_013556	Transferase that aids in the generation of new purine nucleotides from degraded DNA.
Hsp70	heat shock 70kD protein	NM_010478	Heat shock protein that aids in protein folding and cellular stress response.
Pgk1	phosphoglycerate kinase 1	NM_008828	A highly conserved transferase involved in glycolysis that catalyzes the formation of ATP.
Ppia	peptidylprolyl isomerase A	NM_008907	Gene that codes for peptidylprolyl isomerase A, a protein that catalyzes the folding of proteins.
Sdha	Succinate dehydrogenase complex, subunit A	BC011301	Gene that codes for a subunit of succinate dehydrogenase and is important in cellular respiration.
Tbp	TATA box binding protein	NM_013684	Protein that binds to the TATA box sequence and aids in transcription initiation.
Ywhaz	Tyrosine 3-monooxygenase /tryptophan 5-monooxygenase activation protein, zeta polypeptide	NM_011740	Codes for a highly conserved protein that helps mediate signal transduction.

**Figure 1:**
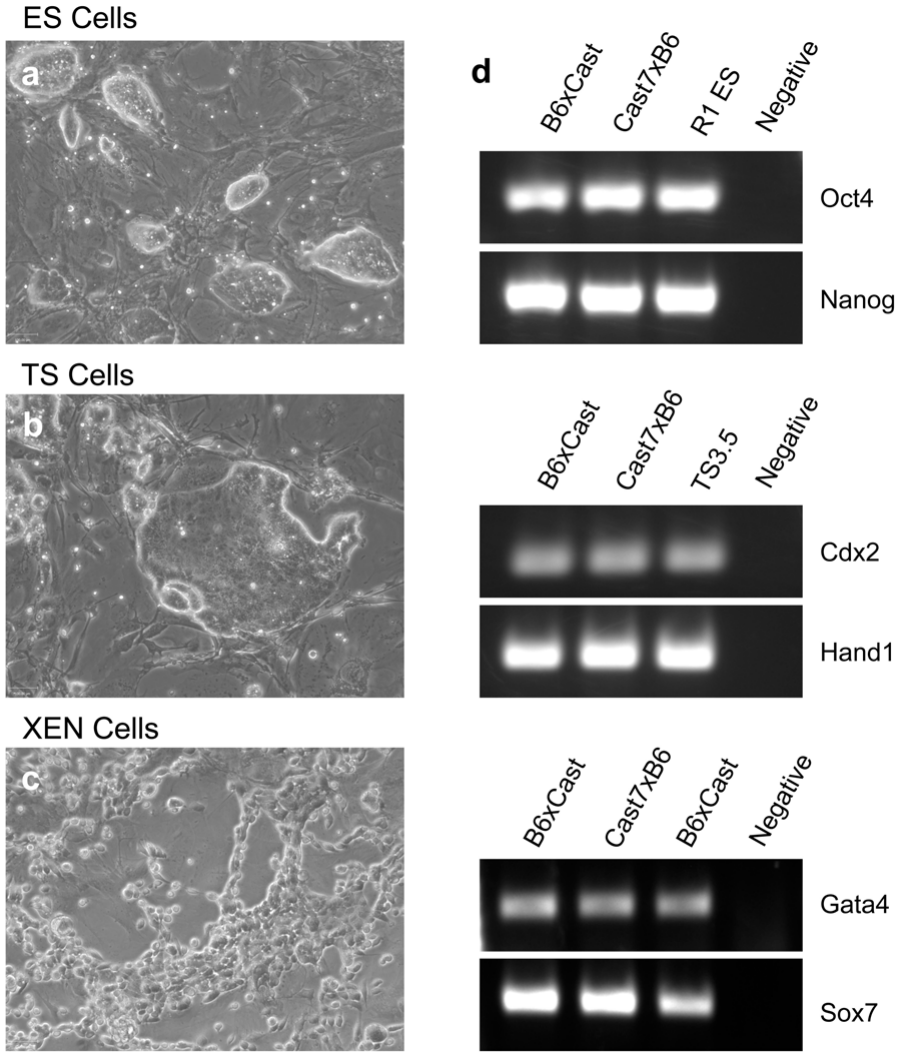
Characteristic cellular morphology and marker gene expression for ES, TS and XEN Stem Cells. a–c Light micrographs of representative ES (a) TS (b) and XEN (c) stem cell lines used in this study. d) Expression of transcription factors characteristic of each of the stem cell lineages.

Previous studies in our laboratory have utilized stem cell lines derived from *Mus musculus castaneus x mus musculus (*C57Black6) F1 embryos. Polymorphisms between these genetic strains allow the examination of mono-allelic patterns of epigenetic marks and gene expression within loci regulated by genomic imprinting [29]. For ES, TS and XEN stem cell analysis we utilized lines derived from F1 embryos of reciprocal crosses between these stains (C57Black6×Castaneous and Cast7×Black6) [11], [29], [30]. For analysis of ES and TS cells we also utilized the previously described R1 ES and TS3.5 lines derived from 129 stain mice [31], [32]. Each of these different lines demonstrated cellular morphology consistent with their cell type and expressed unique cohorts of transcription factors characteristic of their lineage [7], [33] (Figure 1). Cell lines were cultured to 80% confluence, RNA isolated and seeded into five independent qRT-PCR reactions measuring our fourteen candidate genes. Results presented below are the combined analysis of all genetic backgrounds tested.

Of the candidate genes tested *Rn7sk* demonstrated the most robust expression averaging expression levels 125 fold higher than the remaining candidates; which were all readily detectable in each of the cell lines tested. To measure the relative stability of each of the candidate genes between the ES, TS and XEN lines, the CT values for the measured transcripts were compiled and run through the NormFinder, GENorm, and BestKeeper software packages [13]–[15]. Each of these algorithms utilize slightly different methods of estimating both the intra- and the intergroup expression variation, and allow the ranking of candidate genes based on the calculation of a “stability value”. While there was variation amongst the mid-range to least stable genes, all three software packages identified *Pgk1*, *Sdha*, *Tbp* and *H2afz* as the most consistently stable reference genes between ES, TS and XEN stem cells (Table 2). Similar to previous studies by Mamo et al., we observed the classic “housekeeping genes” *Actb*, *Hprt* and to a lesser extent *Gapdh* were comparatively unstable and by our analysis would not be the best choice to normalize qPCR expression levels [19].

**Table 2:** Candidate reference genes ranked in order of their stability.

NormFinder		geNORM	BestKeeper
Rank	Gene Name	Stability Value		Rank	Gene Name	Stability Value		Rank	Gene Name
1	Pgk1	0.012		1	Pgk1	0.121		1	Pgk1
2	Sdha	0.047		2	Sdha	0.132		2	Sdha
3	Tbp	0.054		3	Tbp	0.138		3	H2afz
4	H2afz	0.057		4	H2afz	0.139		4	Tbp
5	Gapdh	0.061		5	Gusb	0.142		5	Gusb
6	Ppia	0.062		6	Gapdh	0.145		6	Ppia
7	Gusb	0.062		7	Ppia	0.145		7	Gapdh
8	Hsp70	0.088		8	Hsp70	0.168		8	Ywhaz
9	Ywhaz	0.088		9	Ywhaz	0.168		9	Hsp70
10	Actb	0.099		10	Actb	0.182		10	Actb
11	B2m	0.107		11	Hprt	0.192		11	Hprt
12	Hk2	0.109		12	B2m	0.192		12	Hk2
13	Hprt	0.109		13	Hk2	0.193		13	B2m
14	Rn7sk	0.128		14	Rn7sk	0.212		14	Rn7sk

a) Stability of the candidate genes between ES, TS and XEN stem cells ranked using the NormFinder, GENorm and BestKeepr software tools.

**Figure 2:**
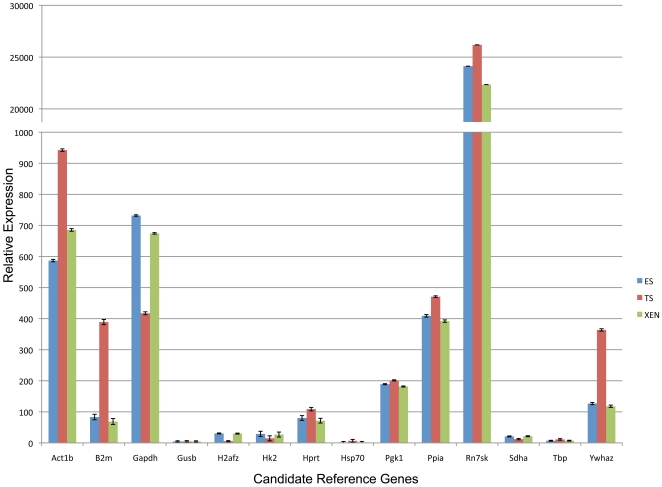
Relative expression of the fourteen candidate genes between all three genetic backgrounds of ES, TS and XEN stem cells. CT values for each measured transcript were normalized to the geometric mean of *Pgk1*, *Sdha* and *Tbp*, and then graphed as relative values using methods described [13], [34], [35]. Error bars represent the standard error of the mean.

We next chose to make pair-wise comparisons between ES and TS, ES and XEN as well as TS and XEN to see which candidates emerged as the most stable in contrasts between any two cell types. A consensus of all three software packages can be seen in table 3. As with the comparisons between all three lines, *Pgk1*, *Sdha*, *Tbp* and *H2afz* remained in the top five most stable genes indicating no one cell type was biasing our analysis and that these five reference genes represent the best normalization controls for qPCR-based analysis of gene expression. Utilizing the geometric mean of *Pgk1*, *Sdha*, and *Tbp* we normalized the CT values for each of the fourteen candidates and graphed their relative expression levels as described previously [13], [34], [35]. As can bee seen in Figure 2, *Rn7sk* is expressed at a drastically higher level than any of the other candidates tested and therefore does not represent a viable reference gene. Similarly, analysis of *Actb*, *B2m*, *Gapdh* and *Ywhaz* all yielded significant differences in measurements of TS cell expression as compared to both ES and XEN cells eliminating their candidacy. Our results indicate normalizing quantitative RT-PCR measurements using the geometric mean CT values obtained for the Pgk1, Sdha and Tbp mRNAs, offers the most reliable method to assess differing patterns of gene expression between the three founding stem cell lineages present within the mammalian preimplantation embryo.

**Table 3:** Consensus of the stability rankings for pair-wise comparisons between the stem cell types.

ES vs. TS	ES vs. XEN	TS vs. XEN
Rank	Gene Name			Rank	Gene Name			Rank	Gene Name
1	Pgk1			1	Pgk1			1	Pgk1
2	Sdha			2	Gapdh			2	Sdha
3	Tbp			3	H2afz			3	H2afz
4	Gusb			4	Tbp			4	Tbp
5	H2afz			5	Sdha			5	Ppia
6	Ppia			6	Gusb			6	Gusb
7	Gapdh			7	Ppia			7	Gapdh
8	Ywhaz			8	Hsp70			8	Ywhaz
9	Hsp70			9	Ywhaz			9	Hsp70
10	Actb			10	Actb			10	Actb
11	Hprt			11	B2m			11	B2m
12	Hk2			12	Rn7sk			12	Hprt
13	B2m			13	Hk2			13	Hk2
14	Rn7sk			14	Hprt			14	Rn7sk

We next sought to determine which of the candidate genes remained the most stable throughout the process of differentiation. Therefore we chose to differentiate our ES and TS cell lines by removal of the key growth factors LIF and FGF4 respectively [6], [36], [37]. To this end ES cells were cultured in LIF - ES cell medium, allowed to form embryoid bodies on untreated plastic dishes and then plated on regular tissue culture plastic to differentiate into fibroblast like cells. Similarly, TS cells were plated on tissue culture treated plastic at low density in FGF4- medium which promoted the formation of TS giant-like cells. We chose not to investigate the process of XEN cell differentiation as reliable protocols for the induction of differentiation have not yet been established. In contrast to both ES and TS cell lines, when XEN cells are plated on plastic many cells simply senesce, while the remainder do not uniformly differentiate into one cell type, thus complicating our analysis.

**Figure 3:**
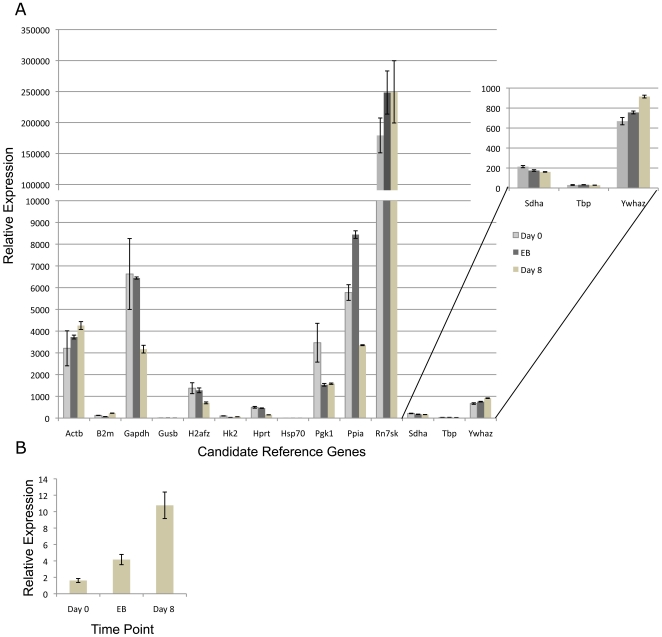
A. Relative expression of the fourteen candidate genes throughout differentiation of all three genetic backgrounds of ES cells examined. CT values for each transcript were measure on Day 0, Day 4 (embryoid body) and Day 8 and were then normalized to the geometric mean of *Sdha Tbp and Ywhaz.* Relative values were determined using methods described previously [13], [34], [35] and graphed. Error bars represent the standard error of the mean. **B.** Increased expression of fibroblast specific protein 1 throughout ES cell differentiation. Error bars represent the standard error of the mean for three independent replicates.

**Figure 4:**
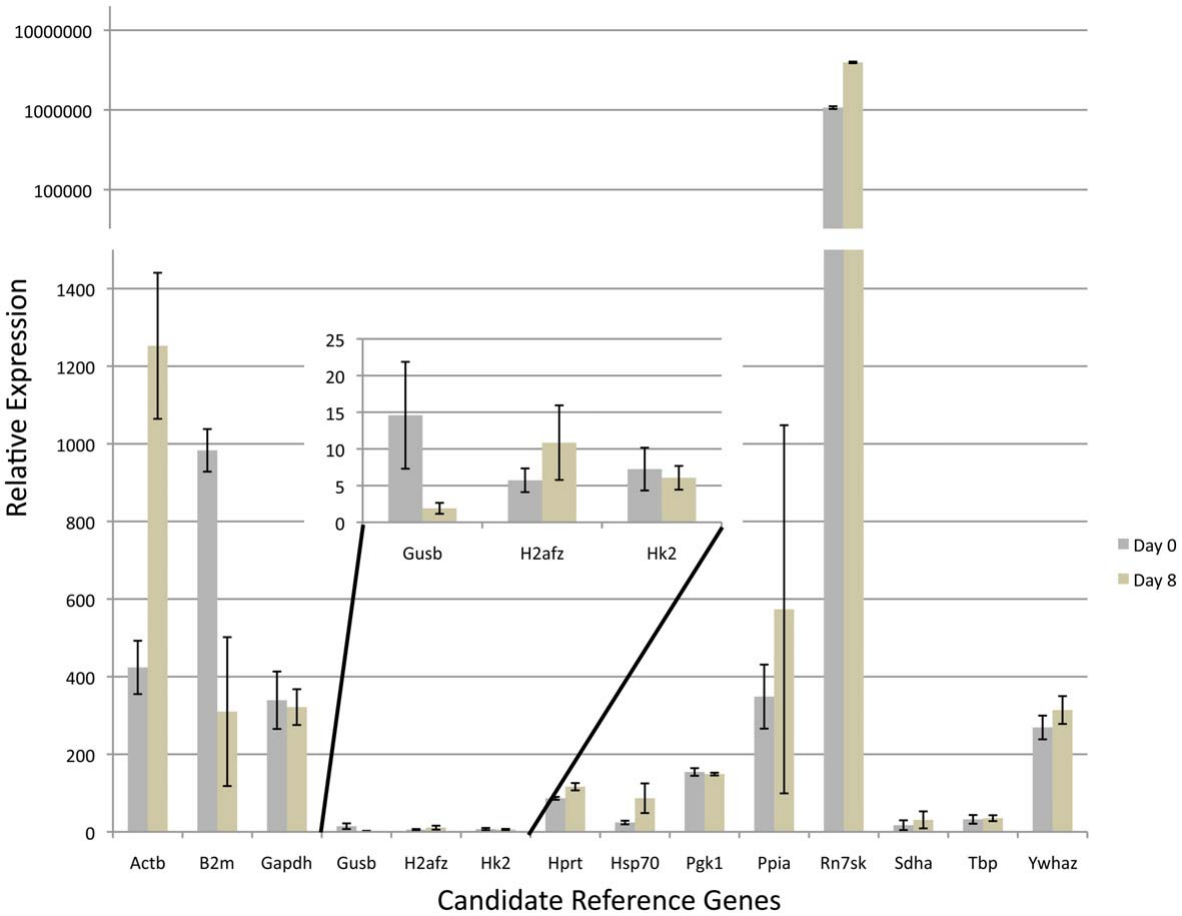
Relative expression of the fourteen candidate genes throughout differentiation of all three genetic backgrounds of TS cells. CT values for each transcript were measured on Day 0 and Day 8 and were then normalized to the geometric mean of *Ywhaz, Pgk1* and *Hk2.* Relative values were determined using methods described previously [13], [34], [35] and graphed. Error bars represent the standard error of the mean. Note that the top third of the graph is in an exponential scale.

RNA samples were collected from ES cells on Day 0, Day 4 (embryoid body) and Day 8 and RNA seeded into five independent qPCR reactions measuring each of the fourteen candidate genes. Using a similar experimental design as described above, we identify Sdha, Tbp and Ywhaz as the three most stable transcripts (Table 4). To examine relative changes in gene expression, we utilized the geometric mean of these three most stable candidates to normalize CT values and graphed the relative expression of all fourteen candidate genes though ES cell differentiation (Figure 3a). We then chose to examine the expression of the cell lineage marker fibroblast-specific protein-1 (FSP-1) which is active in fibroblasts but not in epithelium, mesangial cells or embryonic endoderm [38]. In accordance with previous studies this maker demonstrated increasing expression in differentiating cell cultures, indicating our three candidate genes provided a valid reference point (Figure 3b) [39], [40]. In contrast, transcripts encoding *Pgk1*, *H2afz*, *Ppia* (*Cyclophillin*) and *Gapdh* all demonstrate a significant down-regulation and therefore are not suitable reference genes for this experimental time course. Similar to results reported by Willems et al., examining ES cell differentiation induced by both DMSO and Retinoic acid, we also identify *B2m* and *Hprt* as among the most unstable transcripts [18]. Using similar methodologies, we identified the *Ywhaz*, *Pgk1* and *Hk2* transcripts as the most stable during TS cell differentiation (Table 5). After applying the geometric mean of these three candidates to normalize CT values we observed massive changes in transcripts encoding *Actb*, *B2m* and *Rn7sk* (Figure 4). Previous studies have identified increased actin mobilization as a key feature of trophectoderm stem cell differentiation, validating our identified reference genes [41]. Taken together our data indicate *Sdha*, *Tbp* and *Ywhaz* and *Ywhaz*, *Pgk1* and *Hk2* represent the most stable of our fourteen candidate reference genes for use as qPCR normalization controls during ES and TS cell differentiation respectively.

**Table 4:** Candidate reference genes ranked in order of their stability throughout ES cell differentiation using the NormFinder, GENorm and BestKeepr software tools.

NormFinder		geNORM	BestKeeper
Rank	Gene Name	Stability Value		Rank	Gene Name	Stability Value		Rank	Gene Name
1	Sdha	0.033		1	Sdha	0.005		1	Sdha
2	Tbp	0.033		2	Tbp	0.005		2	Tbp
3	Ywhaz	0.038		3	Ywhaz	0.015		3	Ywhaz
4	H2afz	0.039		4	Gusb	0.017		4	Gusb
5	Gusb	0.039		5	Actb	0.017		5	H2afz
6	Actb	0.04		6	H2afz	0.017		6	Hsp70
7	Hsp70	0.04		7	Hsp70	0.018		7	Actb
8	Gapdh	0.045		8	Gapdh	0.024		8	Gapdh
9	Ppia	0.047		9	Ppia	0.026		9	Ppia
10	Hk2	0.05		10	Hk2	0.027		10	Hk2
11	Pgk1	0.051		11	Pgk1	0.028		11	Hprt
12	Hprt	0.054		12	Hprt	0.033		12	Pgk1
13	Rn7sk	0.057		13	Rn7sk	0.034		13	Rn7sk
14	B2m	0.058		14	B2m	0.036		14	B2m

**Table 5:** Candidate reference genes ranked in order of their stability throughout TS cell differentiation using the NormFinder, GENorm and BestKeepr software tools.

NormFinder		geNORM	BestKeeper
Rank	Gene Name	Stability Value		Rank	Gene Name	Stability Value		Rank	Gene Name
1	Ywhaz	0.095		1	Ywhaz	0.018		1	Ywhaz
2	Pgk1	0.096		2	Pgk1	0.029		2	Pgk1
3	H2afz	0.102		3	Hk2	0.036		3	Hk2
4	Hk2	0.104		4	H2afz	0.04		4	H2afz
5	Hprt	0.11		5	Hprt	0.042		5	Hprt
6	Tbp	0.111		6	Tbp	0.044		6	Tbp
7	Actb	0.112		7	Actb	0.044		7	Sdha
8	Sdha	0.122		8	Ppia	0.048		8	Actb
9	Hsp70	0.127		9	Sdha	0.06		9	Ppia
10	Ppia	0.128		10	Hsp70	0.061		10	Hsp70
11	Gusb	0.133		11	Gusb	0.065		11	Gusb
12	Gapdh	0.153		12	Gapdh	0.093		12	Gapdh
13	B2m	0.183		13	B2m	0.12		13	B2m
14	Rn7sk	0.202		14	Rn7sk	0.24		14	Rn7sk

## Discussion

Analysis of gene expression using qPCR has become the corner stone to nearly every facet of the biological sciences. However, despite numerous studies demonstrating the importance of careful selection and validation of appropriate reference genes, several studies continue to emerge utilizing inappropriate methods of qPCR normalization [13]–[15], [18], [20], [21], [42]. A recent survey of the literature identified the single use of either Actb or Gapdh to normalize expression data in the vast majority of qPCR based studies without any form of validation to ensure their experimental stability [13]. In this study we sought to identify the most stable and appropriate reference genes for studies contrasting patterns of gene expression between the three founding stem cell lineages present within the mammalian preimplantation embryo. From a list of fourteen commonly utilized reference genes we identify *Pgk1, Sdha* and *Tbp* as the most suitable reference genes and further find compelling evidence to suggest that both Actb and *Gapdh* are not suitable normalization controls for these experiments.

Of the top three candidates to emerge from our analysis two are components of pathways controlling cellular respiration. *Pgk1* - *phosphoglycerate kinase 1* is the seventh step of glycolysis and *Sdha* - *Succinate dehydrogenase* or *succinate-coenzyme Q reductase* is an enzyme complex that binds to the inner mitochondrial membrane and is an essential component of both the citric acid cycle and electron transport chain [43], [44]. One potential weakness of our top three candidates is that although *Pgk1* and *Sdha* are components of distinct pathways, they are both components of cellular respiration leaving the possibility that an experimental condition that impacts metabolic processes would significantly alter these normalization controls. The third and fourth candidates to emerge from our analysis were *Tbp* and *H2afz* respectively. *Tbp* is a central component of the RNA polymerase II pre-initiation complex and *H2afz* is an essential component of chromatin structure which is hypothesized to play a role in chromosome organization and stability [45]–[48]. The third and fourth candidates are truly functionally distinct from both each-other and from pathways controlling cellular respiration. As such, where experimental design permits we would recommend normalizing CT values to the geometric mean of all four of these reference genes to improve experimental rigor. However, when we incorporated this strategy we did not observe any meaningful changes in relative gene expression (data not shown).

The first differentiation event during mammalian embryogenesis is the formation of the epiblast, trophectoderm and primitive endoderm which go on to give rise to the three founding embryonic lineages. Stem cells derived from each of these lineages represent an excellent model system to study mammalian development and understand crucial aspects of stem cell biology necessary in developing regenerative therapies. Analysis of gene expression using qPCR will undoubtedly play a pivotal role in deciphering the cellular and molecular properties that define these different cell types. In these analysis, the identification of stable reference genes is an essential prerequisite to accurately interpreting experimental data. Using three independent, highly referenced and validated statistical methods, our analysis of fourteen potential candidate reference genes identify *Pgk1, Sdha* and *Tbp* as the most stable reference genes with which to normalize qPCR data. We believe these three genes will serve as excellent reference controls examining the basis for the differing developmental and epigenetic properties unique to embryonic, trophectoderm and extraembryonic endoderm stem cells.

## Materials and Methods

### Stem Cell Culture

Primary ES cells, TS cells, and XEN cells were derived from either 129 strain (R1 ES cells, [31] TS 3.5) [32] B6×CAST or CAST7×B6 F_1_ embryos [11] as previously described [5]–[7], [11]. Briefly, ES cultures were maintained in DMEM (Sigma, St. Lousi MO. Cat# D5671) supplemented with 50 µg/ml Penicillin/Streptomycin (Invitrogen, Carlsbad CA.), 100 µm β-mercaptoethanol, 1X LIF, (Sigma, St. Lousi MO.) 2 mM L-Glutamine, 1X MEM non-essential amino acids (Invitrogen, Carlsbad CA.) and 15% Hyclone ES grade fetal bovine serum (Fisher Scientific, Pittsburgh PA.). TS and XEN cell cultures were maintained as described (Kunath et al., 2005; Tanaka et al., 1998) using RPMI (Sigma, St. Lousi MI. Cat# R0883) supplemented with 50 µg/ml Penicillin/Streptomycin, 1 mM Sodium Pyruvate (Invitrogen, Carlsbad CA.), 100 µm β -mercaptoethanol, 1 µg/ml Heparin (Sigma, St. Lousi MO), 2 mM L-Glutamine, 1X FGF basic, 1X FGF4 (R&D Systems) and 20% Hyclone ES grade FBS. Cells were grown on a Mytomycin C (Sigma) treated feeder mouse fibroblast layer. For studies examining ES cell differentiation, sub-confluent cultures were dissociated with 1X trypsin (Accutase - Millipore Billerica, MA) and plated on non-tissue culture treated petri dishes in ES cell medium lacking LIF for four days and subsequently plated on 10 cm tissue culture treated dishes to differentiate into fibroblast like cells. To differentiate TS cells we followed methods described previously [32].

### RNA Isolation and Reverse Transcription

Cultured cells were grown to 80% confluence, washed twice in warm PBS, and dissociated with 1X trypsin (Accutase - Millipore Billerica, MA). Cells were spun down, washed once in cold PBS, then RNA isolated using Trizol (Invitrogen, Carlsbad CA.) according to the manufacturer's protocol. One µg of purified total RNA was treated with amplification grade DNaseI (Invitrogen) according to the manufacturer's protocol, and 250 ng RNA seeded into a reverse transcription reaction using the SuperScriptII system (Invitrogen) by combining 1 µl random hexamer oligonucleotides (Invitrogen), 1 µl 10 mM dNTP (Invitrogen), 11 µl RNA plus water. This mixture was brought to 70°C for 5 minutes then cooled to room temperature. SuperScriptII reaction buffer, DTT (Invitrogen) and SuperScriptII were then added according to manufacturer's protocol and the mixture was brought to 25°C for 5 minutes, 42°C for 50 minutes, 45°C for 20 minutes, 50°C for 15 minutes then 70°C for five minutes.

### Real-Time PCR Amplification

Real-time PCR analysis of mRNA levels was carried out using the DyNAmo Flash SYBR Green qPCR Mastermix (Fisher Scientific, Pittsburgh PA.) following the manufacturer's instructions. Reactions were performed on a StepOnePlus Real Time PCR system (Applied Biosystems, Foster City CA.). DNA primer information is available in Table 6. Analysis of Real Time PCR Data

**Table 6:** Description and sequences of the primers used in the in the analysis of both the candidate reference genes and lineage specific transcription factors.

Primer Name	Sequence	Tm	Amplicon Size	Reference
mH2afz Sen	GCGCAGCCATCCTGGAGTA	60		Mamo et al. 2007
m2afz Asen	CCGATCAGCGATTTGTGGA	60	202	
mGAPDH Sen	TGACGTGCCGCCTGGAGAAA	60		Mamo et al. 2007
mGAPDH Asen	AGTGTAGCCCAAGATGCCCTTCAG	60	98	
mHPRT1 Sen	CTGGTGAAAAGGACCTCTCGAA	60		Mamo et al. 2007
mHPRT1 Asen	CTGAAGTACTCATTATAGTCAAGGGCAT	60	117	
mPPIA Sen	CGCGTCTCCTTCGAGCTGTTTG	60		Mamo et al. 2007
mPPIA Asen	TGTAAAGTCACCACCCTGGCACAT	60	150	
mYWHAZ Sen	TTGATCCCCAATGCTTCGC	60		This study
mYWHAZ Asen	CAGCAACCTCGGCCAAGTAA	60	88	
mSDHA Sen 2	GCTCCTGCCTCTGTGGTTGA	60		This study
mSDHA Asen 2	AGCAACACCGATGAGCCTG	60	134	
mB2M Sen	CCGCCTCACATTGAAATCCA	60		This study
mB2M Asen	TCGATCCCAGTAGACGGTCTTG	60	199	
mGUSB Sen 2	GGCTGGTGACCTACTGGATTT	60		This study
mGUSB Asen 2	TTGGCACTGGGAACCTGAAGT	60	134	
mTBP Sen	GAAGAACAATCCAGACTAGCAGCA	60		This study
mTBP Asen	CCTTATAGGGAACTTCACATCACAG	60	127	
mPGK1 Sen	CTGACTTTGGACAAGCTGGACG	60		This study
mPGK1 Asen	GCAGCCTTGATCCTTTGGTTG	60	110	
mHexokinase II Fwd	CCCTGTGAAGATGTTGCCCAC	60		Allen et al.,2004
mHexocinase II Rev	TGCCCATGTACTCAAGGAAGT	60	251	
mActb Fwd	TGGTGGGTATGGGTCAGAAG	57		Allen et al.,2004
mActb Rev	GGTCATCTTTTCACGGTTGG	57	269	
mhsp70 Fwd	ACGTGGCCTTCACCGACACC	57		Allen et al.,2004
mhsp70 Rev	CGATCTCCTTCATCTTCGTC	57	270	
mRn7sk Fwd	ATTGATCGCCAGGGTTGATT	60		Allen et al.,2004
mRn7sk Rev	CGGGGAAGGTCGTCCTCTTC	60	123	
mOct4 Fwd	ATGGCTGGACACCTGGCTTC	60		Golding et al., 2010
mOct4 Rev	GGTCGGCACAGGGCTCAGA	60	337	
mCDX2 Fwd	GCAGTCCCTAGGAAGCCAAGTGA	60		Strumpf et al., 2005
mCDX2 Rev	CTCTCGGAGAGCCCAAGTGTG	60	162	
mGATA4 Fwd	CCTCTATCACAAGATGAACGGC	60		Golding et al., 2010
mGATA4 Rev	CACTGCTGCTGCTGCTGCTA	60	356	
mNanog Fwd	GCACTCAAGGACAGGTTTCAGA	60		Golding et al., 2010
mNanog Rev	GGTGGAGTCACAGAGTAGTTCAGG	60	578	
mSox7 Fwd	CGCCGCCCGCCGTCCCCCGA	60		Golding et al., 2010
mSox7 Rev	CACCCCTGTCCTCCTTCTCC	60	399	
mHand 1 Fwd	GATGCTGCCCCAGATTTCCCT	60		Golding et al., 2010
mHand 1 Rev	CCCTTTTCCGCTTGCTTTCG	60	388	
mFSP1 Fwd	GGCAAGACCCTTGGAGGAG	60		This study
mFSP1 Rev	CCTTTTCCCCAGGAAGCTAG	60	212	

The measured CT (Cycle Threshold) values for each sample were complied and the stability of each of the fourteen reference genes analyzed using the GENorm, NORMFinder and BESTKeeper software tools; which have been described in detail elsewhere [13]–[15]. Once suitable reference genes were identified, the geometirc mean CT values of the best three candidate genes were calculated for each individual sample and used to normalize expression levels using the ▵▵CT method described previously [13], [34], [35]. These normalized values were averaged and the standard error of the mean calculated and graphed using Excel.
